# A compendium of mitochondrial molecular characteristics provides novel perspectives on the treatment of rheumatoid arthritis patients

**DOI:** 10.1186/s12967-023-04426-7

**Published:** 2023-08-22

**Authors:** Qi Wang, Qi-Chao Gao, Qi-Chuan Wang, Li Wu, Qi Yu, Pei-Feng He

**Affiliations:** 1 School of Basic Medical Sciences, Shanxi Medical University, Taiyuan, China; 2 Shanxi Key Laboratory of Big Data for Clinical Decision Research, Taiyuan, China; 3 Department of Anesthesiology, Shanxi Provincial People’s Hospital (Fifth Hospital) of Shanxi Medical University, Taiyuan, China; 4 School of Management, Shanxi Medical University, Taiyuan, China

**Keywords:** Rheumatoid arthritis, Mitochondrial proteins, Immune microenvironment, Unsupervised machine learning, Stratification

## Abstract

Rheumatoid arthritis (RA) is an autoimmune disease that exhibits a high degree of heterogeneity, marked by unpredictable disease flares and significant variations in the response to available treatments. The lack of optimal stratification for RA patients may be a contributing factor to the poor efficacy of current treatment options. The objective of this study is to elucidate the molecular characteristics of RA through the utilization of mitochondrial genes and subsequently construct and authenticate a diagnostic framework for RA. Mitochondrial proteins were obtained from the MitoCarta database, and the R package limma was employed to filter for differentially expressed mitochondrial genes (MDEGs). Metascape was utilized to perform enrichment analysis, followed by an unsupervised clustering algorithm using the ConsensuClusterPlus package to identify distinct subtypes based on MDEGs. The immune microenvironment, biological pathways, and drug response were further explored in these subtypes. Finally, a multi-biomarker-based diagnostic model was constructed using machine learning algorithms. Utilizing 88 MDEGs present in transcript profiles, it was possible to classify RA patients into three distinct subtypes, each characterized by unique molecular and cellular signatures. Subtype A exhibited a marked activation of inflammatory cells and pathways, while subtype C was characterized by the presence of specific innate lymphocytes. Inflammatory and immune cells in subtype B displayed a more modest level of activation (Wilcoxon test P < 0.05). Notably, subtype C demonstrated a stronger correlation with a superior response to biologics such as infliximab, anti-TNF, rituximab, and methotrexate/abatacept (P = 0.001) using the fisher test. Furthermore, the mitochondrial diagnosis SVM model demonstrated a high degree of discriminatory ability in distinguishing RA in both training (AUC = 100%) and validation sets (AUC = 80.1%). This study presents a pioneering analysis of mitochondrial modifications in RA, offering a novel framework for patient stratification and potentially enhancing therapeutic decision-making.

**Supplementary Information:**

The online version contains supplementary material available at 10.1186/s12967-023-04426-7.

## Introduction

 Rheumatoid arthritis (RA) is a heterogeneous and prevalent autoimmune inflammatory arthritis [1], leading to a rise in the number of disabled life years attributed to RA worldwide. However, these trends exhibit regional and national variations. Furthermore, RA is an autoimmune disease with an unknown etiology, and past risk factors include respiratory exposure, genetics, intestinal health, oral health, gender, lifestyle, and habits [2].

Currently, Nonsteroidal anti-inflammatory drugs [3], Glucocorticoids [4, 5], and Disease-Modifying Anti-Rheumatic Drugs (methotrexate, sulfasalazine, minocycline, hydroxychloroquine, and azathioprine) are commonly utilized as the primary pharmacological interventions for managing patients with RA These drugs exert their therapeutic effects through immunosuppressive and anti-inflammatory mechanisms [6–9]. Despite the wide range of treatment options available to RA patients, the current standard treatment regimen is associated with a multitude of adverse effects [10]. In the realm of research, the utilization of big data has the potential to unveil novel (sub-) phenotypes in unsupervised analyses, thereby enhancing precision in medical interventions through the facilitation of innovative targeted therapeutic strategies. Dana E Orange et al. conducted comprehensive analyses of patient samples, leading to the identification of three distinct subtypes of rheumatoid arthritis, with strong associations observed between these subtypes and disease activity [11]. Additionally, Rodrigo Cánovas et al. [12] discovered different subtypes of juvenile idiopathic arthritis, which has significantly contributed to the advancement of our understanding of this disease. Therefore, it is essential to understand RA subtypes and their molecular characterizations in order to better select patients and develop individualized therapy based on phenotypes and molecular signatures.

The disruption of mitochondrial homeostasis has been implicated in the development of RA [13–15]. The imbalance of the endostatin environment resulting from mitochondrial impairment plays a crucial role in the pathology of RA [16]. In the context of RA, METTL3 is responsible for mediating inflammatory responses by activating the NF-κB pathway and facilitating FLS activation [17]. The heightened expression of SIRT4 promotes the secretion of TNF-a and IL-6, thereby expediting the process of bone destruction in individuals with osteoarthritis [18, 19]. Moreover, PTEN Methylation has been found to promote inflammation and the activation of fibroblast-like synoviocytes in Rheumatoid Arthritis [20]. Both mitochondrial metabolism and immune-inflammation are significant pathogeneses of RA. However, their interplay in RA remains unexplored and necessitates further investigation.

This study employed unsupervised clustering methods to identify different subtypes in patients with RA based on mitochondrial gene expression profiles from whole blood. The subtypes were thoroughly characterized using cellular, molecular, and clinical features to gain a deeper understanding of the underlying biological mechanisms. The identified characteristic genes were then applied to independent groups of RA patients to evaluate the therapeutic outcomes of conventional triple Infliximab and anti-TNF. Additionally, machine learning was utilized to develop a diagnostic tool based on the identified features. This study aims to provide a reference for clinical precision treatment and early diagnosis of RA patients.

## Materials and methods

### Processing of RA gene expression data

The Gene Expression Omnibus (GEO) database furnished microarray gene expression data for rheumatoid arthritis samples, with a comprehensive account of the study design, data preprocessing, and data interpretation for the six microarray datasets (GSE110169, GSE93272, GSE58795, GSE15258, GSE37107, and GSE68215 in Additional file 1: Table S1). Several biologic agents were included, namely: Infliximab (GSE58795), anti-TNF (GSE15258), rituximab (GSE37107), methotrexate/abatacept (GSE68215). Drug information was extracted from medical records. Additionally, microarray datasets GSE110169 and GSE93272 were segregated into training and test datasets. To mitigate background noise and normalize quantiles for microarray data, we retrieved raw files in ‘CEL’ format and employed the Affy and Simpleaffy packages for robust multiarray averaging.

### Differentially expressed mitochondrial genes: screening and function and pathway enrichment analysis

The present study employed the Mitocarta 3.01 database to identify gene sets that are associated with mitochondria, with a specific focus on the 1,136 unique human mitochondrial genes [21]. The R package limma was utilized to filter differentially expressed genes that are linked to mitochondria between samples of individuals with RA and healthy control samples. False-positive outcomes were corrected using the false-discovery rate (FDR). The criteria for identifying Mitochondria-associated differentially expressed genes (MDEGs) were an adjusted p-value of less than 0.05 and a log fold change (logFC) of greater than 0.32. To ascertain the enrichment of pathways, a Metascape analysis was executed for GO and KEGG pathways, wherein functional pathways with a p < 0.05 were deemed significantly enriched [22]. Pearson correlation coefficients were employed to scrutinize gene expression correlations.

### Clustering of Mitochondria-related expression-driven subgroups in RA

To gain further insights into molecular subtype heterogeneity within MDEGs profiles associated with RA, the R package ConsensuClusterPlus was utilized to perform hierarchical agglomerative clustering using the ‘km’ method, which is based on Euclidean distance, The parameter settings were as follows: maxK = 6, reps = 1000, pItem = 0.8, pFeature = 1, clusterAlg="km”, distance="euclidean”. The “km” option performs kmeans clustering directly on a data matrix, with items and features resampled. This process was repeated 1000 times to ensure clustering stability [23]. The optimal cluster allocation was determined through the utilization of a cumulative distribution function (CDF). Principal component analysis (PCA) was employed to visualize the differences between subtypes. The identification of differentially expressed genes (MDEGs) was conducted across the three subtypes.

### Characterization of RA subtypes based on cellular, molecular, and clinical characteristics

The present study assessed immune cell infiltration in patients with RA through the utilization of the ‘Xcell’ R package, which facilitated the computation of the enrichment of 64 immune genes [24]. Additionally, the immune function of three subgroups of participants was determined via single-sample gene set enrichment analysis (ssGSEA) [25]. Pathways linked to RA were curated based on literature references and GSEA outcomes, and gene sets were sourced from the KEGG and Reactome databases. The Wilcoxon test was employed to estimate enrichment scores among three subtypes of cells, and statistical significance was determined accordingly.

### Diagnostic gene screening and diagnostic model construction 

Two distinct approaches, LASSO (Least Absolute Shrinkage and Selection Operator) and SVM-RFE (Support Vector Machine-Recursive Feature Elimination), were utilized for screening diagnostic genes [26]. Parameters of LASSO were set as follows: family = “binomial”, alpha = 1, lambda = NULL. Parameters of SVM were set as follows: f functions = rfFuncs, method="repeatedcv”, number = 5, repeats = 3, verbose = FALSE, returnResamp="final”, allowParallel = TRUE. The identification of biomarkers was based on the convergence of the two machine learning algorithms. A diagnostic model was developed through the application of logistic regression analysis, support vector machines, and random forest. Parameters of three machine learning models were set as follows: method="repeatedcv”, number = 10, repeats = 10, classProbs = TRUE, summaryFunction = twoClassSummary, allowParallel = TRUE, and method="glm”, method="svmLinear”, and method=” rf”, metric="ROC”. The pROC package was employed to calculate the area under the ROC curve (AUC) to assess the predictive efficacy of the identified biomarkers.

### Correlation analysis of the identified biomarkers

The CIBERSORT algorithm was utilized to investigate immune-cell infiltration [27], and the correlation between biomarkers and immune cells was analyzed.

### Statistical analysis

All statistical analyses were conducted using R software (version 4.0.3). The Wilcoxon test was employed to compare the differences in pathways between two groups (clusterA-clusterB, clusterB-clusterC, clusterA-clusterC) using the R package ggpubr. The differences in response to treatment between three groups (clusterA, clusterB, and clusterC) using the Fisher test. The statistical significance was defined as a p-value less than 0.05.

## Results

### MDEGs acquisition and functional enrichment

**Fig. 1:**
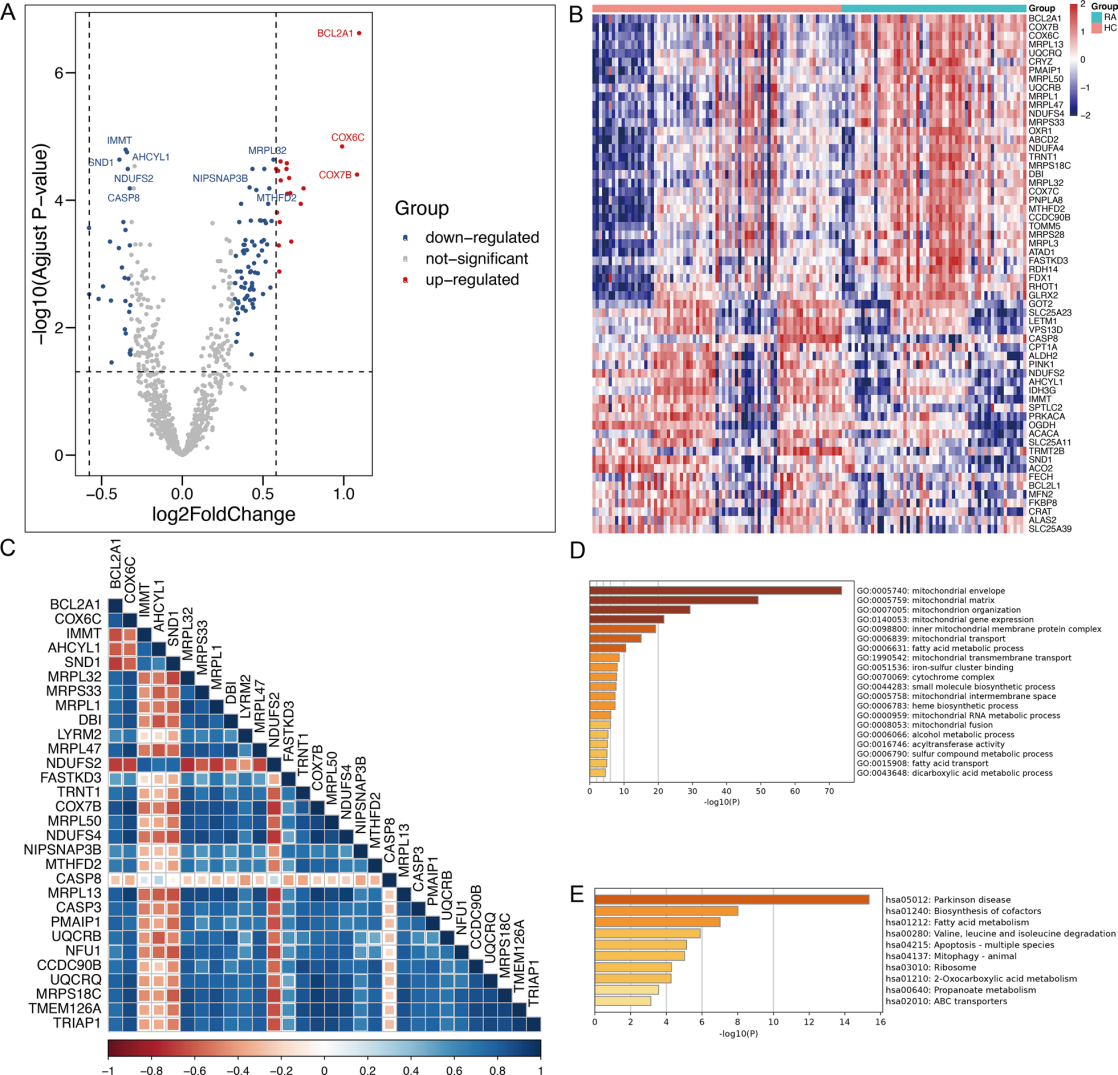
Identification of differentially expressed mitochondrial genes between patients with RA and healthy controls. **A–B** The volcano and heatmap plot of differentially expressed genes between patients with RA and HCs. **C** Correlation heatmap for all 30 mitochondrial genes in RA patients. **D**–**E** GO enrichment and KEGG analyses of 118 differentially expressed mitochondrial genes

To identify the disease-specific differentially expressed Mitochondria-related genes in between RA and HC groups, the limma package was utilized to filter MDEGs between RA and HC groups. In GSE110169, a total of 118 MDEGs were identified from both RA and HC samples (Fig. 1A, B). The Spearman correlation analysis was employed to investigate the relationship among the top 30 genes (Fig. 1C). The results indicated that CASP8, IMMIT, AHCYL1, SND1, and other genes exhibited predominantly negative correlations, while LRP32, MRPS33, and MRPL1 showed mainly positive correlations. GO analysis revealed that these MDEGs were significantly enriched in mitochondrial envelopes, mitochondrial matrix, and mitochondrion organization (Fig. 1D). Additionally, KEGG analyses revealed significant enrichment in the biosynthesis of cofactors and fatty acid metabolism (Fig. 1E). These results suggest a connection between RA and mitochondrial metabolism.

### Clustering of MitoCarta gene expression-driven RA subgroups

**Fig. 2:**
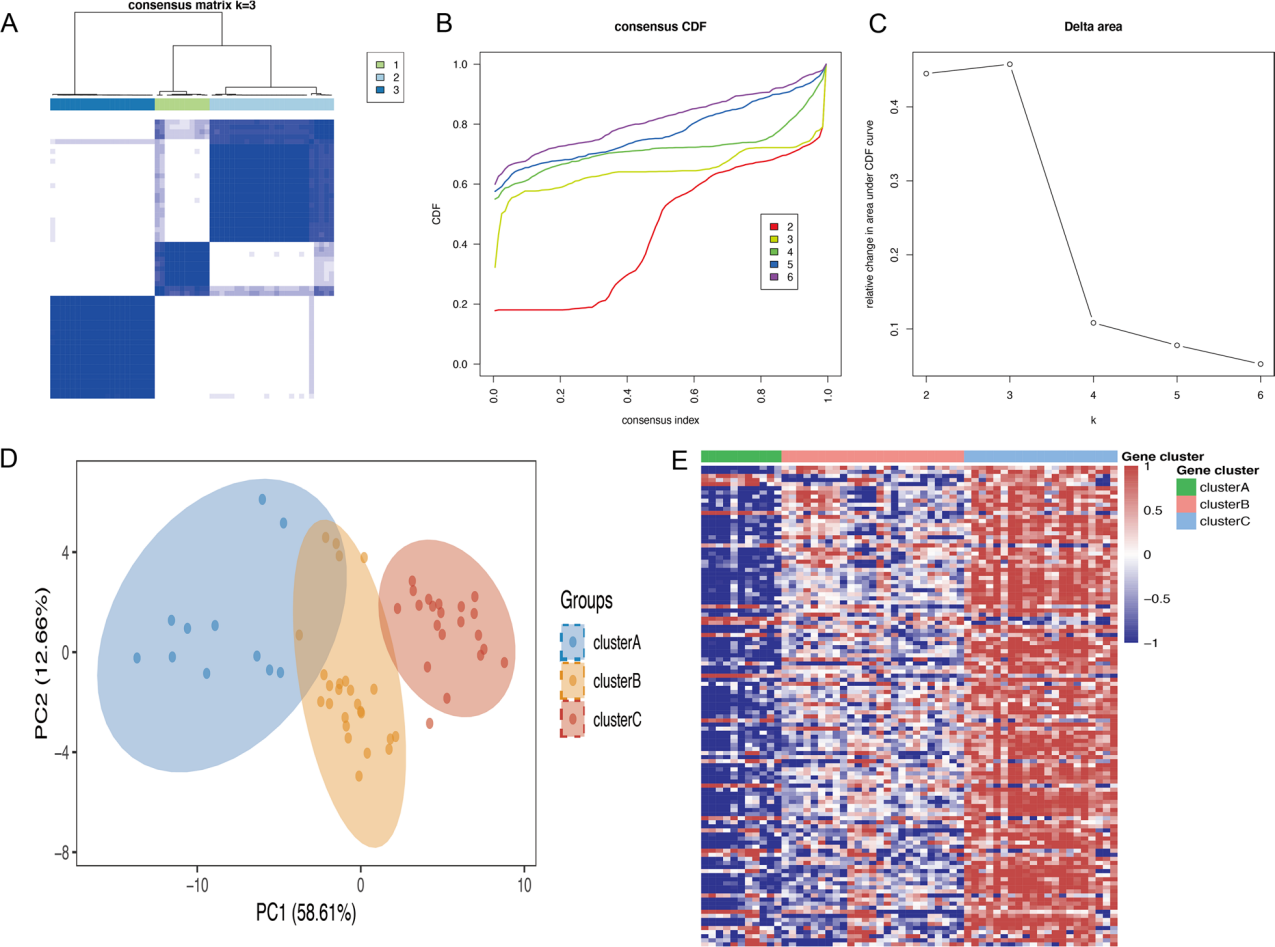
Consensus clustering of RA training cohort. **A** The consensus score matrix for RA samples when k = 3. **B** Consensus clustering cumulative distribution function (CDF) for k = 2–6, which can completely describe the probability distribution of a real random variable. **C** The relative change of CDF Delta area curve for k = 2–6. **D** Principal components analysis for the MDEGs expression profiles showing the stability and reliability of the clustering. **E** The distribution of 118 MDEGs RNA regulators among three clusters

**Fig. 3:**
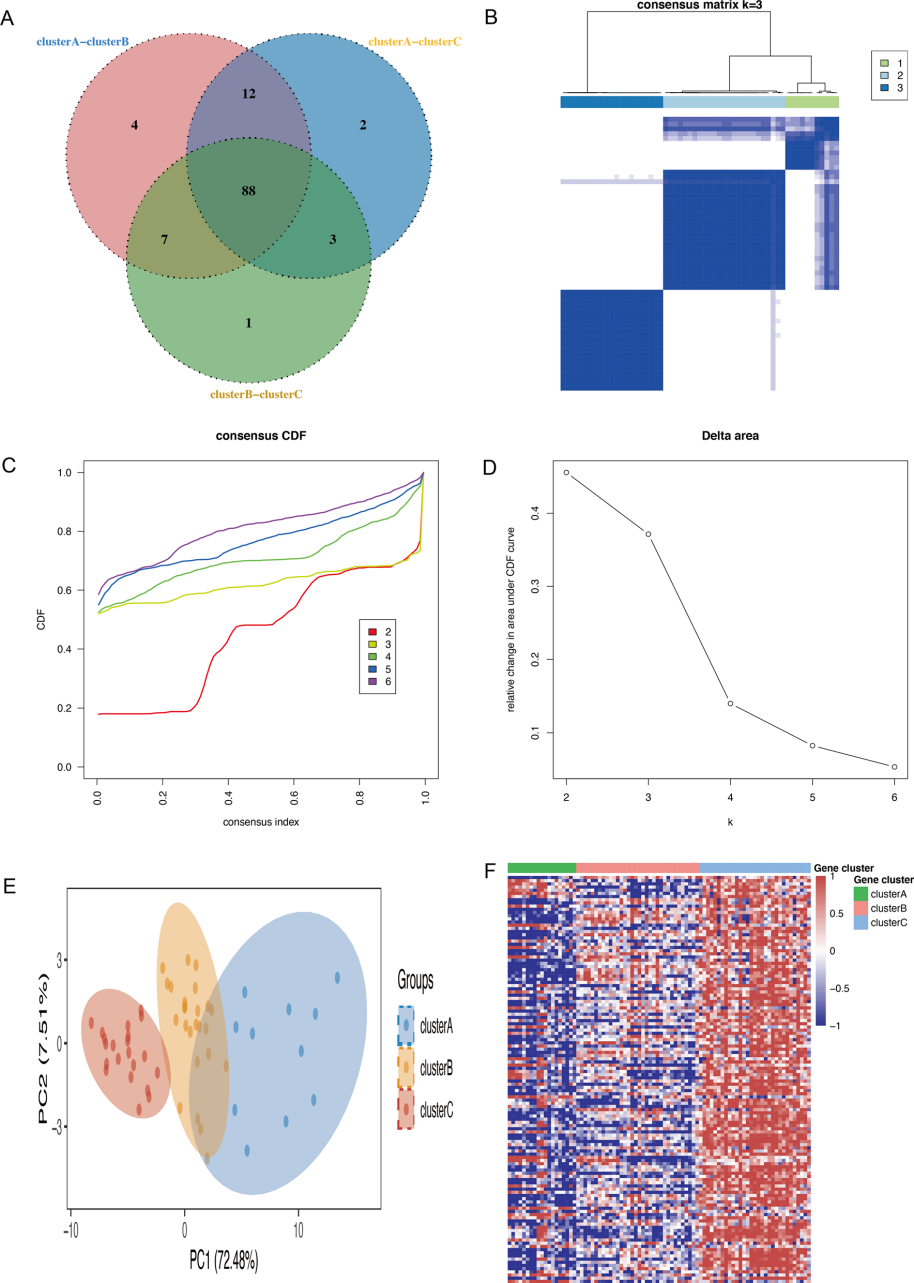
Consensus clustering of RA based on 88 intersection genes. **A** Venn diagram showing the intersection of the MDEGs between three subtypes. **B** The consensus score matrix for RA samples when k = 3. **C** Consensus clustering cumulative distribution function (CDF) for k = 2–6, which can completely describe the probability distribution of a real random variable. **D** The relative change of CDF Delta area curve for k = 2–6. **E** Principal components analysis for the MDEGs expression profiles showing the stability and reliability of clustering. **F** The distribution of 88 MDEGs RNA regulators among three clusters

In order to develop a more comprehensive definition of Mitochondria-related expression-driven subgroups in RA, we conducted 1000 iterations using the ‘ConsensusClusterPlus’ R package with the optimal number of clusters ranging from k = 2 to 6. Based on the CDF values and delta area, we recommend utilizing k = 3 clusters to ensure robust clustering results (Fig. 2A–C). The principal component analyses demonstrated clear segregation among the three subgroups of RA (Fig. 2D), while heatmaps were employed to visualize the differentially expressed genes in the three isoforms (Fig. 2E). Subsequently, an investigation of subtypes was conducted by selecting the 88 genes that were present at the intersection of three subtypes, as depicted in Fig. 3A. The utilization of these 88 genes enabled the identification of three subtypes of RA patients through the same methodology, as illustrated in Fig. 3B and D. The PCA analyses demonstrated a clear differentiation between the three RA subgroups, as shown in Fig. 3E. The MDEGs in the three isoforms were visualized using heatmaps, revealing the presence of three distinct clusters of subtypes: Subtype A (n = 11), Subtype B (n = 25), and Subtype C (n = 21), as presented in Fig. 3F. Overall, these results suggest that stratifying RA patients based on the Mitochondria-related genes in peripheral blood is effective.

### Molecular and cellular characterization of the three subtypes

**Fig. 4:**
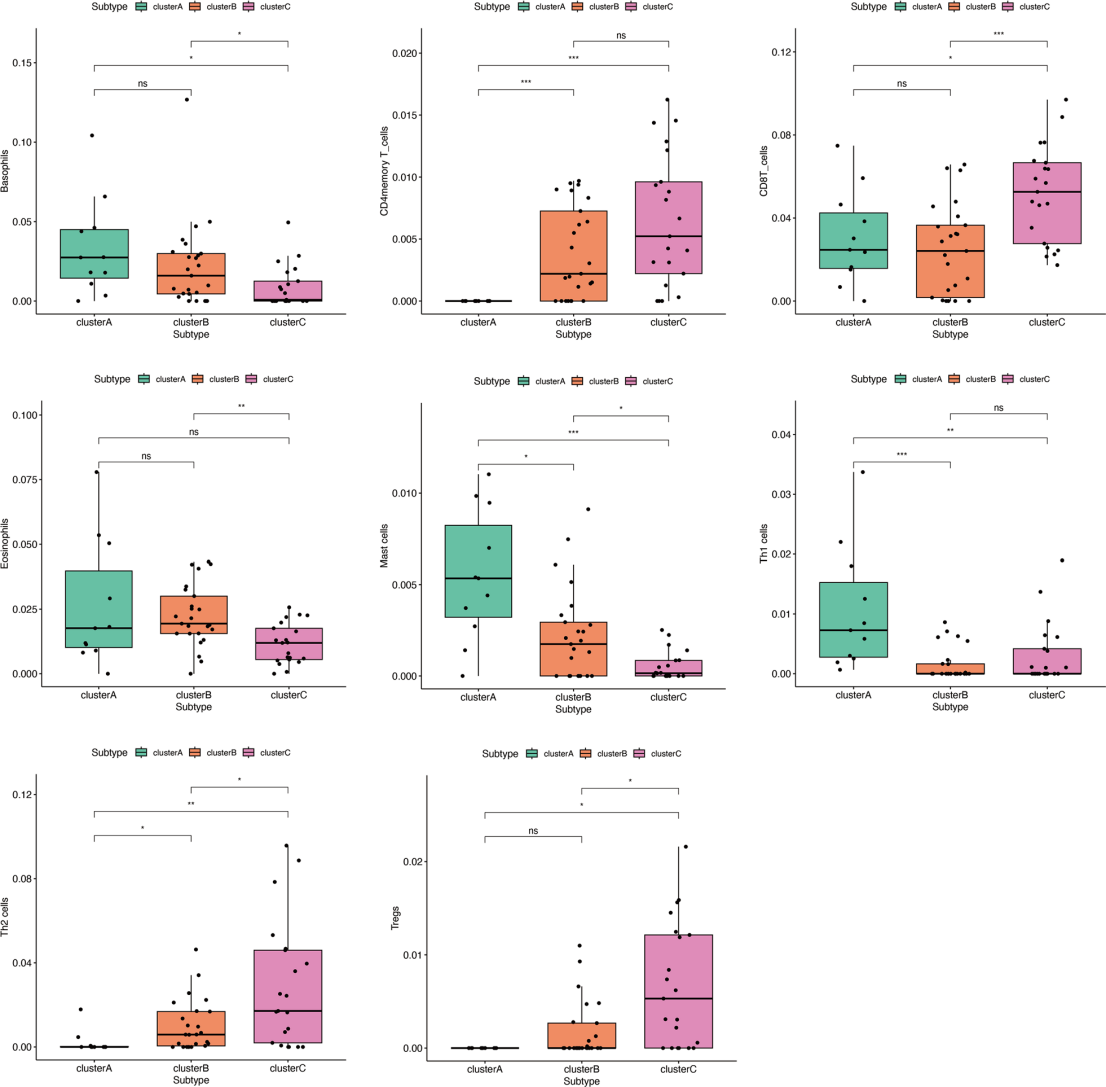
Immune cell characterization of RA subtypes, *p < 0.05; **p < 0.01; ***p < 0.001

**Fig. 5:**
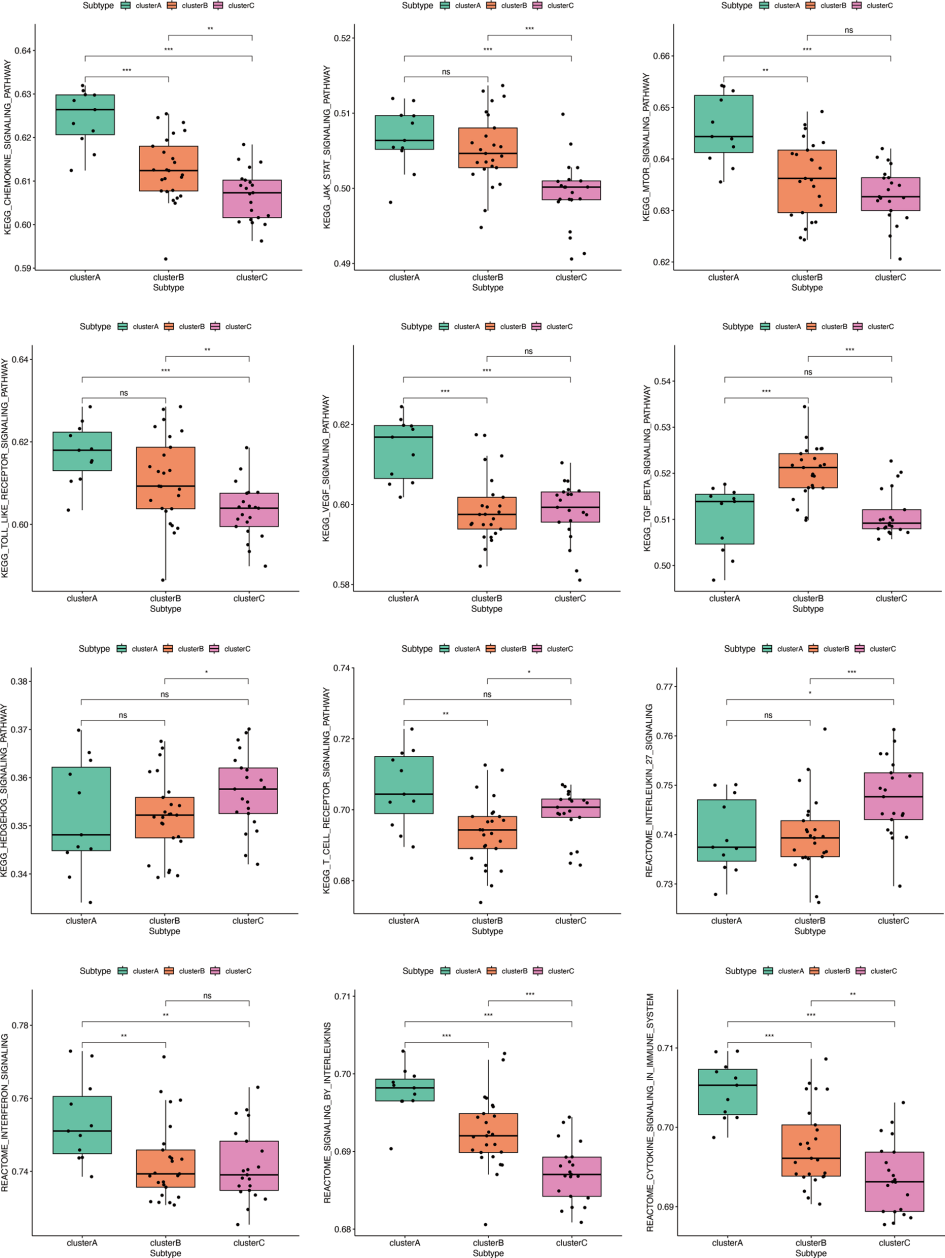
Pathway characterization of RA subtypes, *p < 0.05; **p < 0.01; ***p 0.001

In order to comprehend the molecular attributes and physiological roles of the three resilient subtypes, we conducted an investigation into their prevalence across 64 cell types and immune-related pathways. Notably, Subtype A showed inflammatory cell infiltrates, for example, Basophils, Eosinophils, Mast cells, Th1 cells, and Macrophages. Moreover, subtype A is primarily enriched in Chemokine signaling pathway, JAK stat signaling pathway, mTOR signaling pathway, toll-like receptor signaling pathway, cytokine signaling in immune system, and interferon signaling. Thus, Subtype A was defined as the immune-inflamed type. Subtype C patients had high levels of adaptive immune cells such as activated CD4 + memory T cells, activated CD8 + T cells, and CD2 + T cells. Subtype C was significantly enriched in hedgehog signaling pathway, interleukin_27 signaling, RIG-I-like receptors receptor signaling pathway, and T cell receptor signaling pathway. An innate lymphocyte-rich phenotype was identified in Subtype C. Most inflammatory and immune cells showed modest activation of Subtype B. Subtype B was enriched in TGF beta signaling pathway (Figs. 4 and 5). In conclusion, Subtype A exhibited a marked activation of inflammatory cells and pathways, while subtype C was characterized by the presence of specific innate lymphocytes. Inflammatory and immune cells in subtype B displayed a more modest level of activation.

### Validation of clustering by external cohort

The confirmation of the robustness of the clustering outcomes was established through the utilization of GSE93272. The patients were classified into three subtypes based on the gene expression profiles of 88 MDEGs, namely Subtype A (n = 34), subtype B (n = 39), and subtype C (n = 42) (Additional file 1: Figure S1A–E). Our findings were consistent with the enrichment scores of RA-related pathways and cell subpopulations. Subtype A was characterized by modest activation of inflammatory and immune cells, subtype B was identified as an immune-inflamed type, and subtype C was described as having modest activation of inflammatory and immune cells (Additional file 1: Figures S2, S3).

### Subtypes in response to treatment

**Fig. 6:**
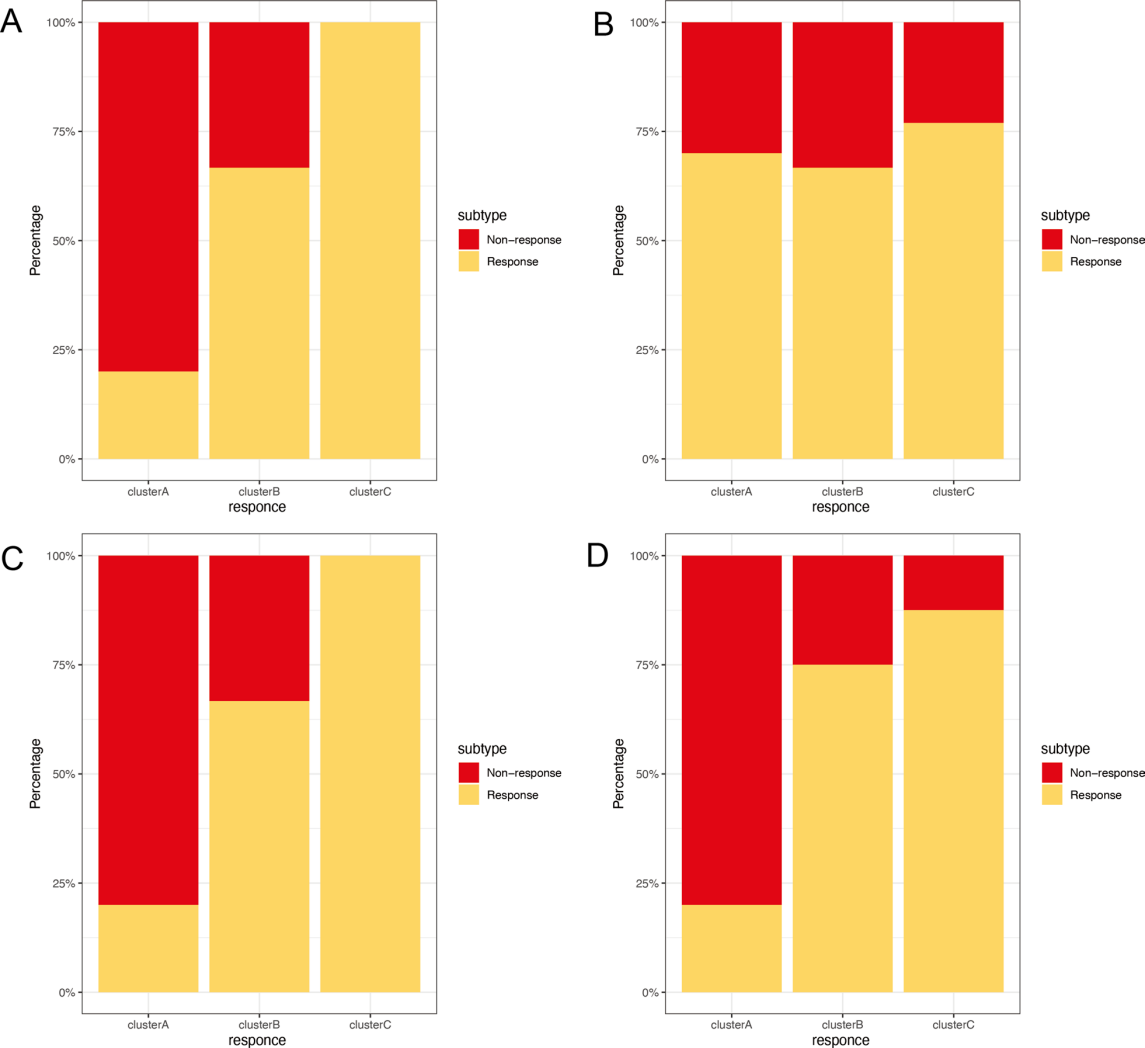
Multiple biologic treatments respond to the RA subtypes. Response: responded to the biologics; non-response: did not respond to the biologics. **A** Response/non-response to Infliximab. **B** Response/non-response to anti-TNF. **C** Response/non-response to rituximab. **D** Response/non-response to methotrexate/abatacept

In order to comprehend the impact of biologic treatment on various subtypes of rheumatoid arthritis (RA), we analyzed four published datasets of RA patients who underwent treatment with Infliximab (GSE58795) (p > 0.05), anti-TNF (GSE15258) (p > 0.05), rituximab (GSE37107) (p > 0.05), methotrexate/abatacept (GSE68215) (p = 0.001). Our findings indicate that subtype C exhibited the highest response rates to all biologics (as illustrated in Fig. 6). Subtype B demonstrated a relatively favorable response. However, due to insufficient sample size, these differences may not have reached statistical significance. Our research suggests that RA can be classified into distinct molecular subtypes, which may impact drug efficacy. Future clinical use of drugs should be considered.

### Construction of diagnostic models

**Fig. 7:**
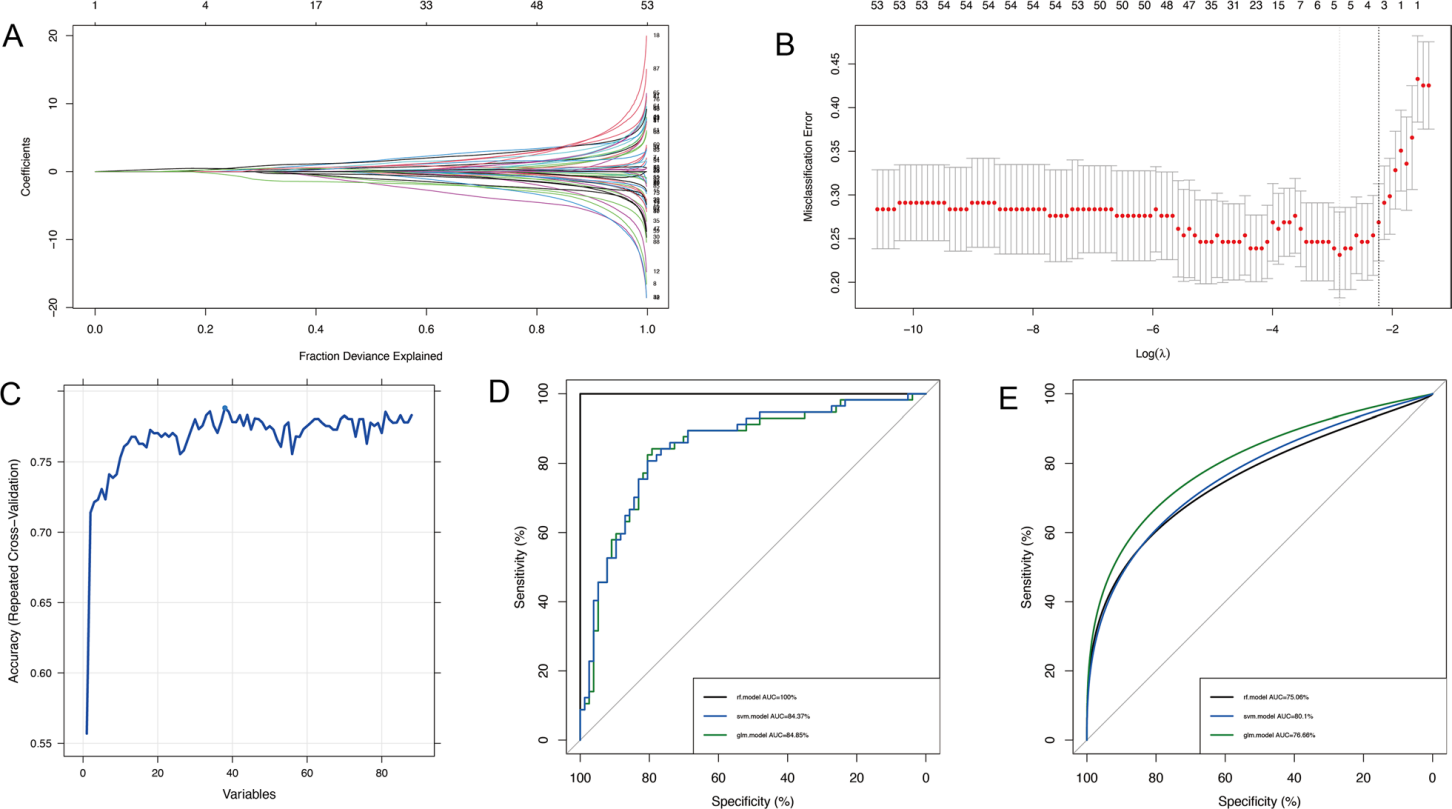
Construction of the RA diagnostic model. **A**–**B** Feature selection proceeded using LASSO. **C** Feature selection proceeded using SVM. **D** ROC curve of RF, glm, and SVM in training set. **E** ROC curve of RF, glm, and SVM in testing set

To screen the key MDEGs of RA, two algorithms were employed to screen potential diagnostic biomarkers, resulting in the identification of five overlapping genes related to diagnosis based on 88 MDEGs, utilizing LASSO logistic regression and SVM algorithms (BCL2A1, MTHFD2, LYRM2, FASTKD3, and ACACA) (Fig. 7A–C). The development of a diagnostic model for RA involved the use of three machine-learning methods. The diagnostic efficacy was subsequently validated using both training (GSE110169) and testing (GSE93272) datasets. The AUCs for RF, SVM, and GL in the training cohort were 100%, 84.37%, and 84.85%, respectively (Fig. 7D). In the testing cohort, the AUCs for RF, SVM, and GL were 75.06%, 80.10%, and 76.66%, respectively (Fig. 7D). The SVM model exhibited a sensitivity of 0.81 and a specificity of 0.71, while the GL model demonstrated a sensitivity of 0.81 and a specificity of 0.68. Similarly, the RF model displayed a sensitivity of 0.77 and a specificity of 0.68. Collectively, these models demonstrated superior predictive performance.

### Correlation analysis biomarkers and infiltrating immune cells

**Fig. 8:**
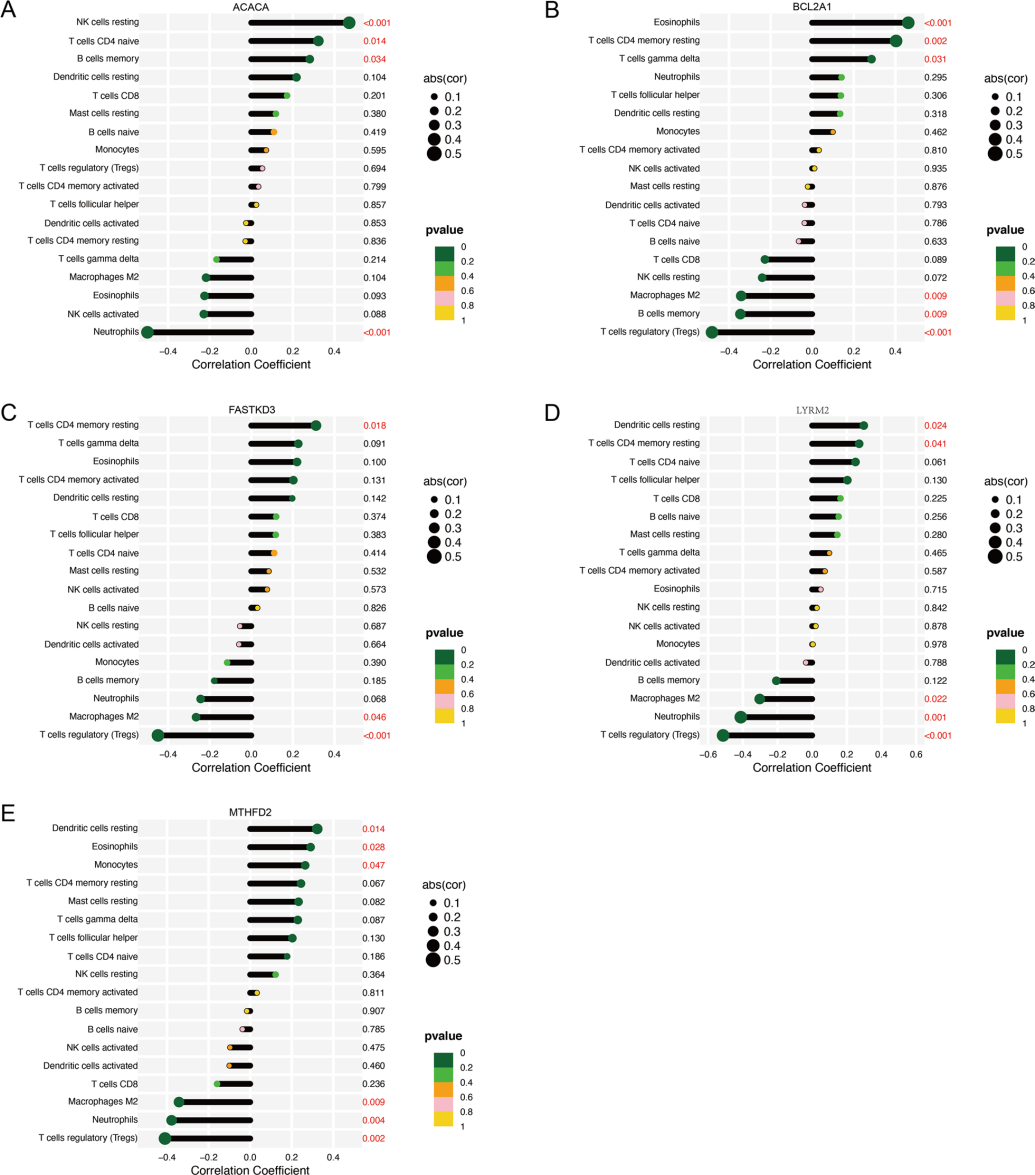
Correlation analysis was shown by spearman correlation analyses between biomarkers and infiltrating immune cells. (**A**) Correlation between ACACA gene and immune cells. (**B**) Correlation between BCL2A1 gene and immune cells. (**C**) Correlation between FASTKD3 gene and immune cells. (**D**) Correlation between LYRM2 gene and immune cells. (**E**) Correlation between MTHFD2 gene and immune cells

The correlation between biomarkers and immune cells was analyzed. ACACA was significantly positively correlated with resting NK cells (P < 0.001), naïve T cells CD4 (P = 0.014), memory B cells (P = 0.034), and significantly negatively correlated with Neutrophils (P < 0.001) (Fig. 8A). BCL2A1 was significantly positively correlated with Eosinophils (P < 0.001), resting T cells CD4 memory (P = 0.002), gamma delta T-cells (P = 0.031), and significantly negatively correlated with Tregs (P < 0.001), memory B cells (P = 0.009), and M2 Macrophages (P = 0.009) (Fig. 8B). FASTKD3 was significantly positively correlated with CD4 memory resting T-cells (P = 0.018), and significantly negatively correlated with Tregs (P < 0.001) and M2 Macrophages (P = 0.046) (Fig. 8C). LYRM2 was significantly positively correlated with resting dendritic cells (P = 0.024) and CD4 memory resting T-cells (P = 0.041), and significantly negatively correlated with Tregs (P < 0.001), Neutrophils (P = 0.001), and Macrophages M2 (P = 0.022) (Fig. 8D). MTHFD2 was significantly positively correlated with resting dendritic cells (P = 0.014), Eosinophils (P = 0.028), and Monocytes (P = 0.047), and significantly negatively correlated with Tregs (P = 0.002), Neutrophils (P = 0.004), and Macrophages M2 (P = 0.009) (Fig. 8E).

## Discussion

This research utilized unsupervised clustering techniques to distinguish three distinct subtypes among patients diagnosed with RA by analyzing mitochondrial gene expression profiles derived from whole blood. Each subtype was linked to unique clinical immune cell fractions and immune-related pathways. Notably, subtype A, characterized as the immune-inflamed type, displayed a transcriptomic signature in inflammatory cells and inflammation and immune-related signaling pathways. In contrast, subtype C, identified as the innate lymphocyte-rich phenotype, exhibited a high degree of enrichment in adaptive immune cells and autoimmune-related pathways. Significantly, subtype B exhibited a modest activation of inflammatory and immune cells, while the three subgroups demonstrated distinct reactions to biologics. A diagnostic model was developed to identify RA patients and prevent the onset of RA.

Patients with rheumatoid arthritis exhibit a unique pattern of mitochondrial regulation, and our investigation has revealed three distinct patterns of RNA modification that are associated with different immune phenotypes. We have confirmed the stability of these subtypes across independent datasets. Subtype C exhibited the highest response rates to Infliximab and anti-TNF. Subtype C is characterized by pronounced inflammatory features, including the activation of CD4 + memory T cells, CD8 + T cells, and CD2 + T cells. TNF is a cytokine with pleiotropic and proinflammatory properties that is primarily produced by activated monocytes and macrophages, and to a lesser extent, by T-lymphocytes [28]. The T cell-mediated response is believed to be particularly crucial in inducing TNF secretion by synovial macrophages [29]. Notably, the normalization of T-cell subsets has been observed in rheumatoid arthritis patients who have undergone long-term treatment with anti-TNF or IL-6R blocker therapies [30–32]. Furthermore, anti-TNF therapy has been shown to promote the expansion of regulatory T cells by paradoxically promoting the binding of membrane TNF-TNF-RII in rheumatoid arthritis [33]. Subtype C exhibited significant enrichment in various signaling pathways, including the hedgehog signaling pathway, interleukin_27 signaling, RIG-I-like receptors receptor signaling pathway, and T cell receptor signaling pathway. The hedgehog signaling pathway is a highly conserved pathway that plays a critical role in embryonic development [34, 35]. The study provided evidence that the utilization of chemically modified siRNA (si-S1A3-Chol) that targets the Hedgehog signaling pathway could serve as a promising therapeutic approach for RA44 [36]. Furthermore, the administration of Anti-TNFα treatment was found to reduce the elevated levels of serum Indian Hedgehog in individuals with ankylosing spondylitis and impact the expression of Hedgehog pathway target genes with functional significance [37]. Recent research indicates that IL-27 may play a role in the pathogenesis of RA through various direct and indirect regulatory pathways. Specifically, IL-27 signaling may impact the development of RA by modulating CD4 + T cell differentiation, suppressing monocytes/macrophages and osteoclasts within the joint cavity, disrupting interactions between synovial ectopic lymphoid structures (ELS) and Th17 cells, and regulating inflammation mediated by RA synovial fibroblasts (RA-FLS) [38, 39]. These findings indicate that the hedgehog signaling pathway and interleukin_27 signaling are significantly enriched in the innate lymphocyte-rich subtype, emphasizing the superior outcomes of anti-TNF and Infliximab.

Research has indicated that mitochondrial dysfunction plays a crucial role in the promotion of RA [40, 41]. Mitochondria are essential organelles that produce energy and play a central role in cellular metabolism. Mitochondrial activity influences the differentiation, activation, and survival of immune and non-immune cells, which contribute to the pathogenesis of RA. A machine learning diagnostic model was developed for patients with RA, which demonstrated favorable predictive performance in both the training and validation datasets. BCL2A1, a member of the BCL-2 family, functions as an anti-apoptotic agent by regulating the intrinsic pathway of apoptosis through the control of cytochrome c release from mitochondria [42]. MTHFD2, an enzyme responsible for mitochondrial NADPH production, is essential for overcoming oxidative stress and maintaining redox homeostasis in tumor cells [43]. However, MTHFD2 deficiency can lead to mitochondrial dysfunction [44]. Additionally, MTHFD2 has been found to inhibit PTEN activity, modulate macrophage polarization, and alter macrophage-mediated immune responses [45]. The LYRM-family proteins have been found to perform a diverse range of crucial functions within the mitochondrion, as evidenced by prior research [46–48]. Specifically, LYRM2 has been shown to play a significant role in the integration of the N-module into respiratory chain complex I [49, 50]. Additionally, FASTKD3 has been identified as having two distinct functions: firstly, it modulates the stability of mature mitochondrial mRNAs ND2, ND3, CYTB, COX2, and ATP8/6; and secondly, it promotes COX1 mRNA translation [51]. Furthermore, ACACA, which functions as the rate-limiting enzyme of FAS, acts as a catalyst for the carboxylation of CO2 and the conversion of acetyl-CoA into malonyl-CoA [52]. Notably, ACACA has been found to suppress prostate cancer through the inhibition of mitochondrial potential. In summary, these genes are mainly involved in metabolism processes and affect disease progression.

This study represents the initial attempt to comprehensively examine the correlation between mitochondrial genes and rheumatoid arthritis. By identifying three unique patterns of mitochondrial gene modification, we have gained a deeper understanding of the underlying mechanisms. Furthermore, we have constructed a diagnostic model that exhibits clinical efficacy, which may prove valuable in future investigations of mitochondrial gene modification in RA. Ultimately, these findings have the potential to enhance therapeutic decision-making and improve the accuracy of treatment response prediction. However, it is difficult to refute the fact that this study was subject to certain limitations. The study relied on bioinformatics analysis, and a number of its findings require validation through subsequent experiments and extensive cohorts.

### Supplementary Information

**Additional file 1: Table S1. **Summary information of patients with RA. **Figure S1.** Consensus clustering of RA validation cohort. (A) The consensus score matrix for RA samples when k = 3. (B) Consensus clustering cumulative distribution function (CDF) for k = 2-6, which can completely describe the probability distribution of a real random variable. (C) The relative change of CDF Delta area curve for k = 2-6. (D) Principal components analysis for the MDEGs expression profiles showing the stability and reliability of the clustering. (E) The distribution of 88 MDEGs RNA regulators among three clusters. **Figure S2.** Immune cell characterization of RA subtypes, *p <0.05; **p <0.01;***p < 0.001. **Figure S3.** Pathway characterization of RA subtypes, *p <0.05; **p <0.01;***p 0.001.

## Data Availability

The datasets supporting the conclusions of this article are available in the GEO repository, (https://www.ncbi.nlm.nih.gov/geo/). The names of the repository/repositories and accession number(s) can be found in the Supplementary material.
