# Supplementary material for: A compendium of mitochondrial molecular characteristics provides novel perspectives on the treatment of rheumatoid arthritis patients
Source: J Transl Med. 2023 Aug 22;21:561. doi: 10.1186/s12967-023-04426-7 (PMC10463924; doi:10.1186/s12967-023-04426-7)
Supplement: Supplementary file 1 — Additional file 1: Table S1. Summary information of patients with RA. Figure S1. Consensus clustering of RA validation cohort. (A) The consensus score matrix for RA samples when k = 3. (B) Consensus clustering cumulative distribution function (CDF) for k = 2-6, which can completely describe the probability distribution of a real random variable. (C) The relative change of CDF Delta area curve for k = 2-6. (D) Principal components analysis for the MDEGs expression profiles showing the stability and reliability of the clustering. (E) The distribution of 88 MDEGs RNA regulators among three clusters. Figure S2. Immune cell characterization of RA subtypes, *p <0.05; **p <0.01;***p < 0.001. Figure S3. Pathway characterization of RA subtypes, *p <0.05; **p <0.01;***p 0.001. [file 12967_2023_4426_MOESM1_ESM.docx]

**Table S1: Summary information of patients with RA**

| **Data set** | **Subjects** | **Experiment type** | **Platforms** | **Collection site** | **Reference** | |
| --- | --- | --- | --- | --- | --- | --- |
| **Training set** |  |  |  |  |  |  |
| Accession: GSE110169 | 57 RA vs 77 HC | Expression profiling by array | GPL13667 | Coulter Department of Biomedical Engineering, USA | Yanhua Hu et all. | PMID: 29534336 |
| **Testing set** |  |  |  |  |  |  |
| Accession: GSE93272 | 115 RA 43 HC | Expression profiling by high throughput sequencing | GPL570 | Fujisawa, Norway | Shinya Tasaki  et al. | PMID: 30013029 |
| **Treatment set** |  |  |  |  |  |  |
| Accession: GSE58795 | Infliximab (23 Response vs 7 Non-response) | Expression profiling by array | GPL10379 | Boston, USA | Kenzie D MacIsaac et al. | PMID: 25504080 |
| Accession: GSE15258 | Anti-TNF (53 Response vs 22 Non-response) | Expression profiling by array | GPL570 | Cambridge, USA | Jadwiga R Bienkowska et al. | [PMID: 19699293](https://www.ncbi.nlm.nih.gov/pubmed/19700435) |
| Accession: GSE37107 | Rituximab (8 Response vs 6 Non-response) | Expression profiling by array | GPL6947 | Amsterdam, Netherlands | Alexandre E Voskuyl et al. | PMID: 22540992 |
| Accession: GSE68215 | Methotrexate/Abatacept (17 Response vs 19 Non-response) | Expression profiling by array | GPL4133 | Rouen cedex, France | Derambure C et al. | PMID: 28545499 |


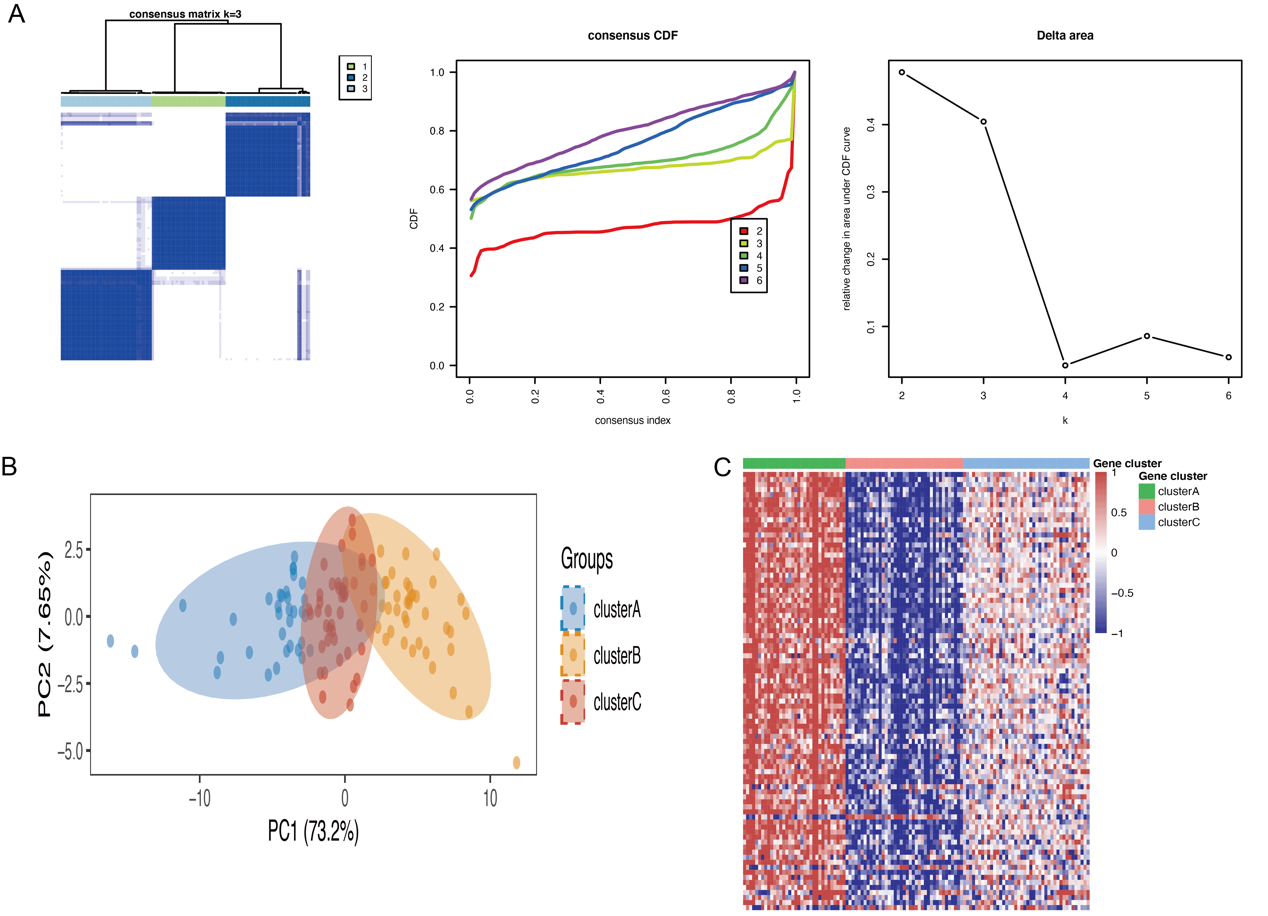


**Additional figure 1 Consensus clustering of RA validation cohort.** (A) The consensus score matrix for RA samples when k = 3. (B) Consensus clustering cumulative distribution function (CDF) for k = 2-6, which can completely describe the probability distribution of a real random variable. (C) The relative change of CDF Delta area curve for k = 2-6. (D) Principal components analysis for the MDEGs expression profiles showing the stability and reliability of the clustering. (E) The distribution of 88 MDEGs RNA regulators among three clusters.


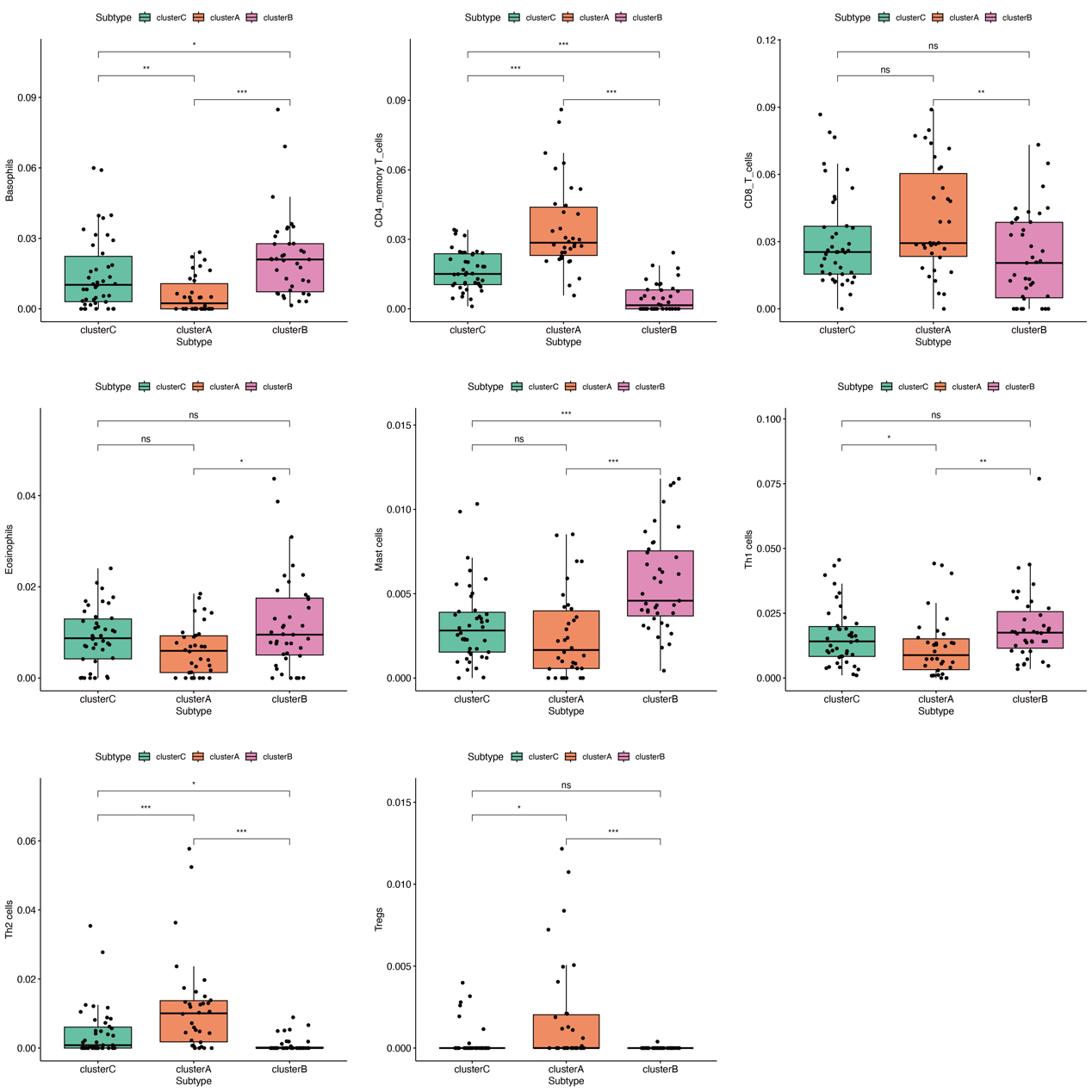


**Additional figure 2 Immune cell characterization of RA subtypes,** **p* <0.05; ***p* <0.01; ****p* < 0.001.


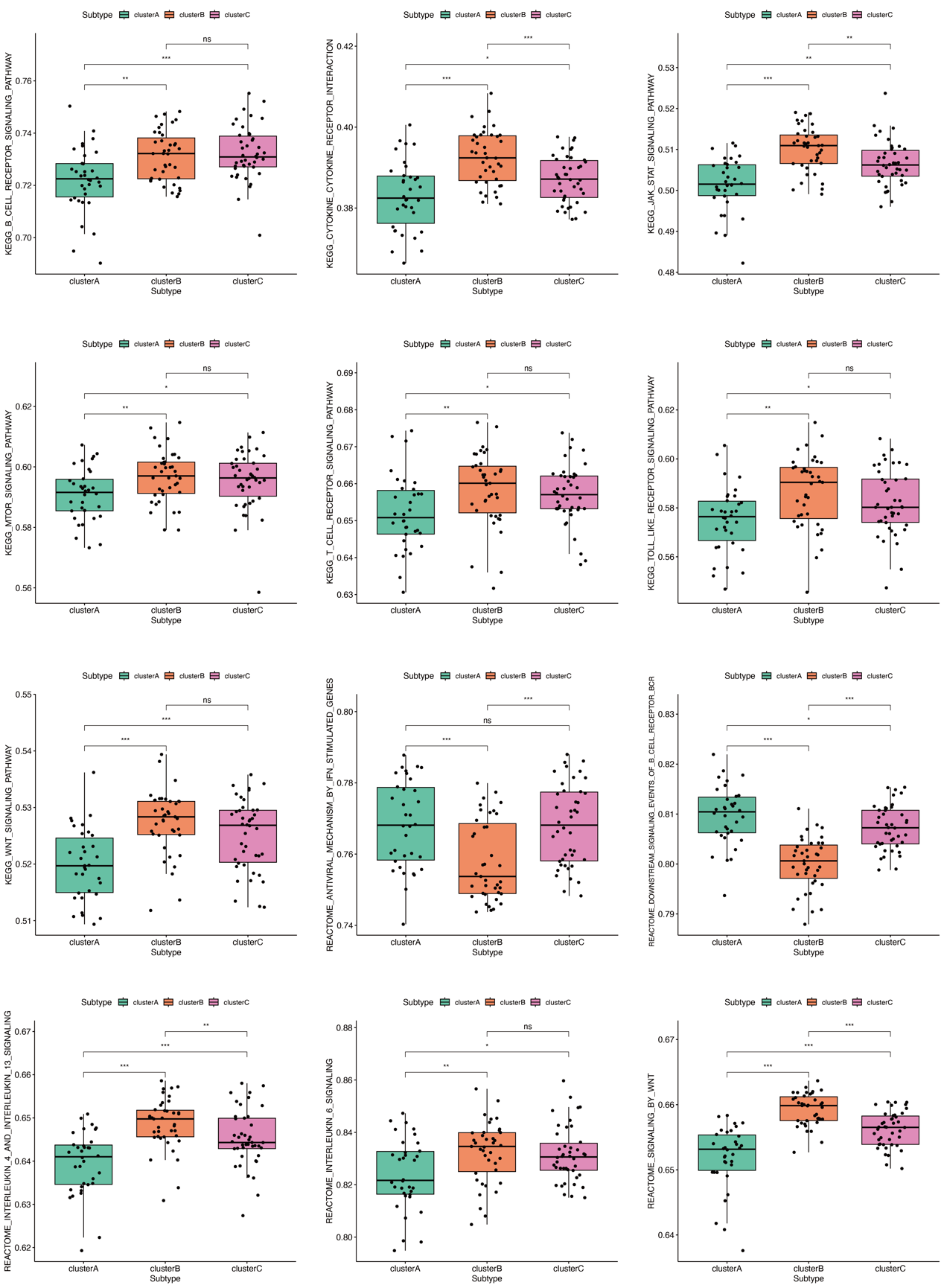


**Additional figure 3 Pathway characterization of RA subtypes,** **p* <0.05; ***p* <0.01; ****p* 0.001.
